# Elevated UHRF1 expression contributes to poor prognosis by promoting cell proliferation and metastasis in hepatocellular carcinoma

**DOI:** 10.18632/oncotarget.14446

**Published:** 2017-01-02

**Authors:** Xincheng Liu, Huohui Ou, Leyang Xiang, Xianghong Li, Yu Huang, Dinghua Yang

**Affiliations:** ^1^ Department of Hepatobiliary Surgery, Nanfang Hospital Affiliated to Southern Medical University, Guangzhou, China; ^2^ The Second Affiliated Hospital of Shantou University Medical College, Shantou, China; ^3^ Department of Laboratory Medicine, Nanfang Hospital Affiliated to Southern Medical University, Guangzhou, China

**Keywords:** HCC, UHRF1, prognosis, proliferation, invasion

## Abstract

Ubiquitin-like with plant homeodomain and ring finger domains, 1 (UHRF1) is overexpressed in a variety of tumor tissues and is negatively correlated with prognosis of patients with cancers, yet so far, a comprehensive study of UHRF1 in hepatocellular carcinoma (HCC) has not been conducted. The present study was designed to explore the expression of UHRF1, associated clinical implications, and its possible functions in HCC. Reverse transcription-polymerase chain reaction and immunohistochemical staining were used to detect UHRF1 expression in HCC specimens including cancerous and noncancerous tissues. Associations of UHRF1 expression with demographic and clinicopathologic features in HCC were analyzed, and the effects of RNA interference of UHRF1 on cell proliferation, cell cycle, apoptosis, and migration were investigated *in vitro* and *in vivo*. UHRF1 mRNA and protein expression were both upregulated and negatively correlated with prognosis in HCC patients. Furthermore, inhibition of proliferation, migration, invasion, and epithelial-mesenchymal transition progression were observed *in vitro* and *in vivo* after UHRF1 knockdown, moreover, G2/M arrest was detected in HCC cells. In conclusion, elevated UHRF1 expression contributes to poor prognosis by promoting cell proliferation and metastasis in HCC.

## INTRODUCTION

Cancer is the leading cause of death since 2010 and a major public health problem in China. In 2015, there were an estimated 4,292,000 newly diagnosed invasive cancer cases and 2,814,000 cancer deaths in China. Liver cancer is the fourth most commonly diagnosed cancer and the third leading cause of cancer death [1]. Based on histological differences, liver cancer can be divided into three types, the most common of which is hepatocellular carcinoma (HCC), which accounts for approximately 90% of primary liver cancers [2, 3]. At present, experts believe that in China hepatitis B is one of the main pathogenic factors in HCC, and our previous work indicated that more than 90% of HCC patients are diagnosed with hepatitis B [4, 5], which can progress to liver fibrosis and cirrhosis, and may ultimately lead to tumorigenesis [6, 7]. Generally, HCC is initially asymptomatic and patients show typical symptoms only once the disease has progressed to the advanced stage.

Advances in biotechnology and imaging technology have enabled diagnosing HCC at an early stage, and developments in surgical techniques and non-surgical treatments have improved the overall survival of patients with HCC [8, 9]. However, our previous follow-up studies showed that recurrence or metastasis within five years is observed in approximately 60% of HCC patients undergoing radical resection [4, 5]. Most researchers think this phenomenon is correlated with biological characteristics of high recurrence and the metastatic potential of HCC [10, 11]. Pathological examination of resected tissues indicated that microvascular tumor thrombus is present in 50% of HCC patients, and regression analysis showed that preoperative microvascular invasion is an independent risk factor for poor prognosis [12, 13]. Therefore, early detection, early diagnosis, and early treatment are key to the management of HCC. After decades of efforts, researchers have found multiple biomarkers and unraveled various signaling pathways in carcinogenesis, and new treatments have emerged accordingly [8, 9]; yet, none of these are satisfactory. Thus, continued efforts at screening for new biomarkers associated with HCC remains necessary.

Ubiquitin-like with plant homeodomain and ring finger domains, 1 (UHRF1), also known as Np95 or ICBP90 [14, 15], is a nucleoprotein associated with tumorigenesis [16, 17]. Researchers found that UHRF1 regulates gene expression and chromatin modification by interacting with DNA methyltransferase 1 and histone deacetylase 1 [18–20], and UHRF1 overexpression drives DNA hypomethylation and oncogenesis [21]. Previous study indicated that UHRF1 is overexpressed in a variety of tumor tissues such as gastric cancer, bladder cancer and HCC, and UHRF1 overexpression is negatively correlated with prognosis of HCC patients [21–26]. Additionally, the proliferative and invasive abilities of tumor cells were significantly inhibited after UHRF1 knockdown, while opposite results were obtained when UHRF1 was upregulated [27–29]. Furthermore, studies have shown that UHRF1 is regulated by microRNAs, and mTOR inhibitors showed an antitumor effect by downregulating UHRF1 [30, 31]. These findings suggest that UHRF1 may be a valuable prognostic biomarker and a promising drug target. To date, a comprehensive study of UHRF1 in HCC has not been conducted. In the present study, associations of UHRF1 mRNA and protein expression in matched HCC tissue specimens with demographic and clinicopathologic features were analyzed, and the effects of RNA interference of UHRF1 on cell proliferation, cell cycle, apoptosis, and migration were investigated in *vitro* as well as *in vivo*.

## RESULTS

### UHRF1 expression is elevated and correlates with poor prognosis in HCC

**Figure 1:**
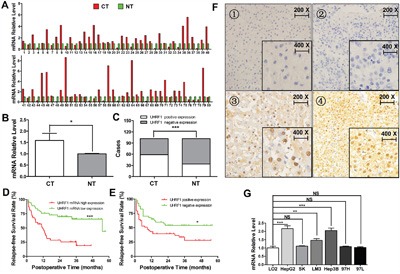
UHRF1 expression is elevated and correlates with poor prognosis in HCC **A** and **B**. Comparison of the expression levels of UHRF1 mRNA (n = 80) between HCC cancerous tissues (CT) and the matched noncancerous tissues (NT); **C**. Comparison of the expression levels of UHRF1 protein between CT and NT (n = 102); **D**. Kaplan-Meier analysis post-operative relapse-free survival time between high and low UHRF1 mRNA expression; **E**. Kaplan-Meier analysis post-operative relapse-free survival time between positive and negative UHRF1 protein expression; **F**. UHRF1 protein is localized in cell nuclei: 1, negative staining in noncancerous tissues; 2, negative staining in cancerous tissues; 3 and 4, positive staining in cancerous tissues. **G**. mRNA expression of UHRF1 in the HCC cell lines and the immortalized hepatic cell line LO2. Columns, mean of three or five independent data; bars, SEM. *P < 0.05, **P < 0.01, ***P < 0.001, NS = not significant.

Previous studies have indicated that UHRF1 is overexpressed in many types of tumors [21–23]. We initially explored UHRF1 mRNA expression using qRT-PCR in a retrospective cohort of 80 HCC samples. UHRF1 mRNA expression was elevated in 67.5% (54/80, *P* < 0.05) of HCC tissues as compared to the matched noncancerous tissues (Figure 1A and 1B). UHRF1 protein expression was detected by IHC staining in 102 pairs of HCC samples. UHRF1 protein was localized in the nucleus (Figure 1F), and the positive expression rate of UHRF1 protein in the cancerous tissues was significantly higher than that in the matched noncancerous tissues (57.8% vs. 32.7%, *P* < 0.05, Figure 1C), which is consistent with the previous finding [25]. Additionally, UHRF1 mRNA expression was especially higher in HepG2, HCCLM3, and Hep3B than that in the transformed hepatocyte cell line LO2 (*P* < 0.05 for each line, Figure 1G). Univariate analysis showed that elevated UHRF1 mRNA and protein expression were prominently associated with clinicopathologic parameters such as tumor size, tumor differentiation, TNM stage, tumor microemboli, tumor metastasis, recurrence, and relapse-free survival time (*P* < 0.05, Tables 1 and 2). Together, these results indicated that elevated UHRF1 expression correlates with malignant clinicopathologic parameters of HCC. Patients with elevated UHRF1 expression had a shorter relapse-free survival time (*P* < 0.05) as indicated by Kaplan-Meier analysis (Figure 1D and 1E), indicating that UHRF1 may be a promising biomarker for predicting outcomes of HCC patients.

**Table 1:** Relationships between UHRF1 mRNA expression and clinical pathological parameters in HCC patients

Clinical characteristics		Cases	high UHRF1 mRNA expression	low UHRF1 mRNA expression	*x*^2^	*P*
Gender	Male	69	34 (49.3%)	35 (50.7%)	0.105	0.745
	Female	11	6 (54.5%)	5 (45.5%)		
Age (years)	<60	71	38 (53.5%)	33 (46.5%)	3.130	0.077
	≥60	9	2 (22.2%)	7 (77.8%)		
AFP^a^ (μg/L)	<400	54	25 (46.3%)	29 (53.7%)	0.912	0.340
	≥400	26	15 (57.7%)	11 (42.3%)		
Cirrhosis	Yes	55	28 (50.9%)	27 (49.1%)	0.058	0.809
	No	25	12 (48%)	13 (52%)		
Tumor number	=1	64	30 (46.9%)	34 (53.1%)	1.250	0.264
	>1	16	10 (62.5%)	6 (37.5%)		
Tumor size (cm)	≤5.0	36	13 (36.1%)	23 (63.9%)	5.051	0.025
	>5.0	44	27 (61.4%)	17 (38.6%)		
Tumor capsule	Yes	69	33 (47.8%)	36 (52.2%)	0.949	0.330
	No	11	7 (63.6%)	4 (36.4%)		
Differentiation	Well	12	3 (25%)	9 (75%)	5.599	0.061
	Moderate	49	24 (49%)	25 (51%)		
	Poor	19	13 (68.4%)	6 (31.6%)		
TNM stage	I+II	38	14 (36.8%)	24 (63.2%)	5.013	0.025
	III+IV	42	26 (61.9%)	16 (38.1%)		
BCLC stage	A	53	20 (37.7%)	33 (62.3%)	12.28	0.002
	B	11	6 (54.5%)	5 (45.5%)		
	C	16	14 (87.5%)	2 (12.5%)		
Micro-tumor embolus	Yes	41	26 (63.4%)	15 (36.6%)	6.054	0.014
	No	39	14 (35.9%)	25 (64.1%)		
Metastasis	Yes	35	26 (74.3%)	9 (25.7%)	14.679	0.000
	No	45	14 (31.1%)	31 (68.9%)		
Relapse*	Yes	41	28 (68.3%)	13 (31.7%)	9.618	0.002
	No	34	11 (32.4%)	23 (67.6%)		
Relapse-free survival time (months)*	<6	18	13 (72.2%)	5 (27.8%)	5.654	0.059
	6≤x<12	11	7 (63.6%)	4 (36.4%)		
	≥12	46	19 (41.3%)	27 (58.7%)		

^a^AFP: α-fetoprotein. *Data are missing for 5 patients.

**Table 2:** Relationships between UHRF1 protein expression and clinical pathological parameters in HCC patients

Clinical characteristics		Cases	UHRF1 protein positive expression	UHRF1 protein negative expression	*x*^2^	*P*
Gender	Male	88	51 (58.0%)	37 (42.0%)	0.003	0.954
	Female	14	8 (57.1%)	6 (42.9%)		
Age (years)	<60	87	51 (58.6%)	36 (41.4%)	0.147	0.702
	≥60	15	8 (53.3%)	7 (46.7%)		
AFP^a^ (μg/L)	<400	71	43 (60.6%)	28 (39.4%)	0.709	0.400
	≥400	31	16 (51.6%)	15 (48.4%)		
Cirrhosis	Yes	75	46 (61.3%)	29 (38.7%)	1.415	0.234
	No	27	13 (48.1%)	14 (51.9%)		
Tumor number	=1	76	42 (55.3%)	34 (44.7%)	0.814	0.367
	>1	26	17 (65.4%)	9 (34.6%)		
Tumor size (cm)	≤5.0	36	13 (36.1%)	23 (63.9%)	10.776	0.001
	>5.0	66	46 (69.7%)	20 (30.3%)		
Tumor capsule	Yes	64	39 (60.9%)	25 (39.1%)	0.675	0.411
	No	38	20 (52.6%)	18 (47.4%)		
Differentiation	Well	27	9 (33.3%)	18 (66.7%)	9.077	0.011
	Moderate	55	37 (67.3%)	18 (32.7%)		
	Poor	20	13 (65.0%)	7 (35.0%)		
TNM stage	I+II	64	32 (50.0%)	32 (50.0%)	4.334	0.037
	III+IV	38	27 (71.1%)	11 (28.9%)		
BCLC stage	A	69	37 (53.6%)	32 (46.4%)	4.266	0.118
	B	16	13 (81.3%)	3 (18.8%)		
	C	17	9 (52.9%)	8 (47.1%)		
Micro-tumor embolus	Yes	51	35 (68.6%)	16 (31.4%)	4.865	0.027
	No	51	24 (47.1%)	27 (52.9%)		
Metastasis	Yes	56	38 (67.9%)	11 (32.1%)	5.107	0.024
	No	46	21 (45.7%)	25 (54.3%)		
Relapse*	Yes	53	35 (66.0%)	18 (34.0%)	4.116	0.042
	No	40	18 (45.0%)	22 (55.0%)		
Relapse-free survival time (months)*	<6	32	25 (78.1%)	7 (21.9%)	8.994	0.011
	6≤x<12	12	6 (50.0%)	6 (50.0%)		
	≥12	49	11 (44.9%)	35 (55.1%)		

^a^AFP: α-fetoprotein. *Data are missing for 9 patients.

### UHRF1 was successfully knocked down in HCC cells

**Figure 2:**
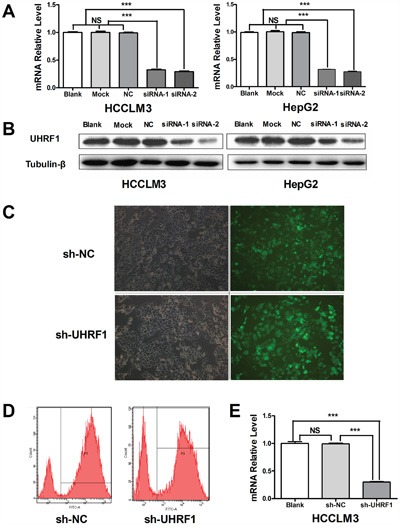
Depletion of UHRF1 expression in HCC cells **A**. The transfection efficiency of UHRF1 siRNAs was assayed by qRT-PCR in HCCLM3 and HepG2 cells after transfected with for 48 h; **B**. The transfection efficiency of UHRF1 siRNAs was assayed by immunoblotting; **C**. UHRF1 depletion efficiency was assessed under an inverted fluorescence microscope after HCCLM3 cells were transfected with shRNAs for 48 h; **D**. The transfection efficiency of UHRF1 shRNAs was confirmed by FACS analysis; **E**. The transfection efficiency of UHRF1 shRNAs was confirmed by qRT-PCR. Columns, mean of three independent data; bars, SEM. ***P < 0.001, NS = not significant.

We further explored the functions of UHRF1 in HCCLM3 and HepG2 because of their relatively high UHRF1expression. SiRNA was used to transiently deplete the expression of UHRF1 in the HCC cells. RT-PCR and western blotting showed that UHRF1 was effectively knocked down at both the mRNA and the protein level after 48 h of transfection (Figure 2A and 2B). ShRNA was used for stable knockdown of UHRF1 in HCCLM3. Stable fluorescent signal was observed after transfection for 96 h (Figure 2C), and flow-cytometric analysis indicated that the transfection efficiency was satisfactory (83.4% in the sh-NC group and 71.8% in the sh-UHRF1 group) (Figure 2D). The mRNA expression level of UHRF1 in sh-UHRF1 cells was significantly decreased as compared to that in sh-NC and non-transfected cells (Figure 2E). Thus, the interference sequences are effective in depleting the expression of UHRF1 in HCC cells.

### UHRF1 downregulation inhibits tumor growth *in vitro* and *in vivo*

**Figure 3:**
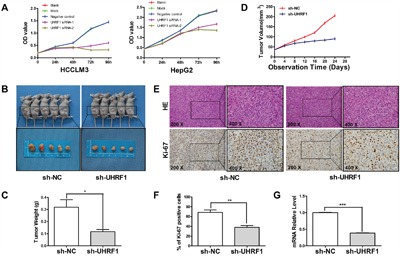
UHRF1 depletion inhibits tumor growth *in vitro* and *in vivo* **A**. HCCLM3 and HepG2 cells were transfected with siRNAs for 48 h followed by CCK-8 assay to detect cell proliferation at 0 h, 24 h, 48 h, 72 h, and 96 h after transfection; **B**. HCCLM3 cells that were transfected with shRNAs were implanted into nude mice through subcutaneous injection, tumor volume differed between the sh-UHRF1 group and the sh-NC group; **C**. tumor weight differed between the sh-UHRF1 group and the sh-NC group; **D**. Tumor growth curves revealed a difference between the sh-UHRF1 group and the sh-NC group; **E** and **F**. H&E and Ki-67 IHC staining of tumor nodules and quantitative analysis; **G**. qRT-PCR analysis of UHRF1 mRNA expression in tumor nodules. Columns, mean of three or five independent experiments; bars, SEM. *P < 0.05, **P < 0.01, ***P < 0.001, NS = not significant.

Sustained proliferative signaling is one of the hallmarks of cancer [32]. Researchers have recently shown that the proliferative ability of cancer cells was inhibited after UHRF1 knockdown [22]. In our study, cell proliferation was significantly restrained after UHRF1 downregulation in the two HCC cell lines (*P* < 0.05 for each line, Figure 3A). To understand the influence of UHRF1 on tumor growth, xenografted models were established in nude mice using lentiviral-mediated stably transfected HCCLM3 cells. The results showed that tumor volumes and weights in the sh-UHRF1 group were obviously lower than those in the sh-NC group (Figure 3B and 3C), and the tumor growth curves showed that UHRF1 silencing significantly inhibited tumor growth (*P* < 0.05, Figure 3D). Furthermore, we carried out H&E staining and IHC staining for Ki-67 in the xenografted tissues. H&E staining showed that atypical cells were obvious in subcutaneous tumors in both groups, and the numbers of Ki-67-positive cells were lower in the sh-UHRF1 than in the control group, which was in accordance with the *in vitro* data (Figure 3E and 3F). qRT-PCR analysis of the tumors indicated that the mRNA expression level of UHRF1 in sh-UHRF1 mice was significantly lower than that in sh-NC mice (*P* < 0.05, Figure 3G). We concluded that UHRF1 may prompt tumor proliferation in HCC.

### UHRF1 knockdown leads to cell cycle arrest but does not induce apoptosis in HCC cells

**Figure 4:**
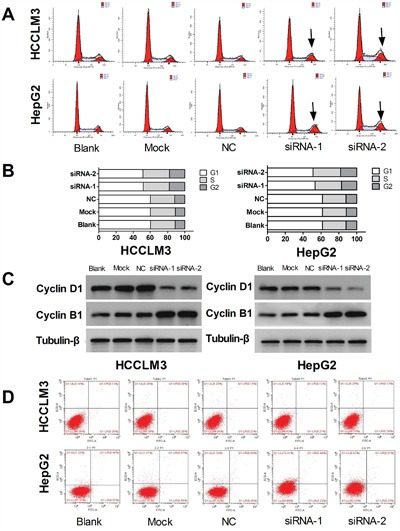
UHRF1 knockdown leads to cell cycle arrest, but does not induce apoptosis in HCC cells **A**. Changes of cell cycle were determined by FACS analysis in HCCLM3 and HepG2 cells after transfected with siRNAs for 48 h; **B**. Quantification of cells in the different cell cycle phases; **C**. Western blot analysis of cyclin expression; **D**. Apoptosis changes were determined by FACS analysis.

It is becoming increasingly apparent that uncontrolled cell division is a defining characteristic of cancer cells, and cyclins play indispensable roles in cell cycle control [33, 34]. To determine whether UHRF1 influences cell cycle, we analyzed cell cycle distribution after UHRF1 knockdown using a cell cycle assay kit. FACS analysis showed that G2/M phase cells increased and G1 phase cells decreased after UHRF1 knockdown in HepG2 and HCCLM3 cells, while there were no obvious changes in the percentage of S cells (*P* < 0.05, respectively, Figure 4A and 4B). Western blot analysis showed that cyclinB1 was upregulated while cyclinD1 was downregulated in UHRF1-silenced cells (Figure 4C). Therefore, we concluded that G2/M phase cell cycle arrest was induced after UHRF1 knockdown.

Apoptosis is an ordered and orchestrated cellular process that occurs in physiological and pathological conditions. At present, cancer is considered one of the scenarios where too little apoptosis occurs, resulting in malignant cells that will not die [35]. To explore the effect of UHRF1 on apoptosis, flow cytometry was used to detect the changes of apoptosis after UHRF1 knockdown in the HCC cells. However, apoptosis was not significantly induced after UHRF1 knockdown in the two cell lines (Figure 4D).

### UHRF1 silencing inhibits migration and invasion in HCC cells, and restrains epithelial-mesenchymal transition (EMT) progression *in vitro* and *in vivo*

**Figure 5:**
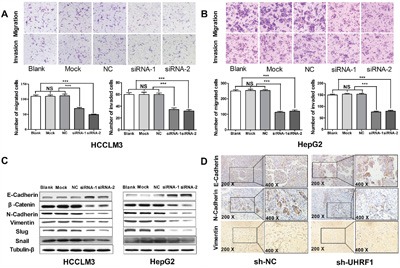
UHRF1 silencing inhibits migration and invasion in HCC cells, and restrains EMT progression *in vitro* and *in vivo* **A**. Transwell migration and invasion assays were carried out in transfected HCCLM3 cells after transfected with siRNAs for 48 h; **B**. Transwell migration and invasion assays were carried out in transfected HepG2 cells after transfected with siRNAs for 48 h; **C**. Western blot analysis of EMT markers in HCC cells; **D**. IHC detection of EMT markers in the tumor nodules. Columns, mean of three independent data; bars, SEM. ***P < 0.001, NS = not significant.

Activating invasion and metastasis is another hallmark of cancer contributing to poor prognosis of cancer patients [32]. To clarify the effect of UHRF1 on cellular migration and invasion abilities after UHRF1 knockdown in HCC cells, transwell assays were used. The results showed that the numbers of migrated and invaded cells decreased significantly (*P* < 0.05 for both) in the two UHRF1 knockdown groups (Figure 5A and 5B) as compared to the control groups. Thus, UHRF1 may exert a pro-metastatic effect on HCC.

EMT has been increasingly recognized to occur during the progression of HCC [36]. To investigate the effect of UHRF1 on EMT progression, EMT-related proteins were detected by western blotting after UHRF1 knockdown. The expression of E-cadherin was upregulated and N-cadherin, vimentin, β-catenin, and snail were downregulated after UHRF1 knockdown (Figure 5C). Additionally, EMT-related proteins were investigated using IHC staining in the xenografted tissues. The results showed that the expression of N-cadherin and vimentin in subcutaneous tumor was significantly lower than that in the sh-NC group, while E-cadherin was upregulated (Figure 5D). Therefore, we consider that UHRF1 may prompt EMT process in HCC.

## DISCUSSION

With the development of science and technology, treatment options for HCC, including radical resection, radiotherapy, chemotherapy, and liver transplantation are increasing. However, the overall prognosis of HCC patients is still not satisfactory [2, 3]. Recently, the molecular targeted agent sorafenib brought hope for patients with HCC; it improved the prognosis significantly in a multicenter, phase 3, double-blind, placebo-controlled trial [37]. However, because of the complex pathogenesis and heterogeneity among different individuals, we found that sorafenib is only effective in a minority of HCC patients in a clinical 21-23, 38], and researchers have studied the expression of UHRF1 in HCC and its clinical significance. Besides, some researchers believed that UHRF1 overexpression drives DNA hypomethylation and promotes HCC, and some researchers suggested UHRF1 overexpression drives HCC by regulating the expression of maternally expressed gene 3 [21, 26]. But there is no comprehensive study on the specific role of UHRF1 in HCC, therefore, further exploration of UHRF1 in HCC is helpful to increase our understanding of the pathogenesis of HCC and to provide a theoretical basis for the treatment of HCC.

Aberrant overexpression of UHRF1 was detected in many types of cancer tissues and was also associated with poor prognosis of bladder patients [39, 40]. To further explore the roles of UHRF1 and its significance in HCC, in this study, we first investigated the expression of UHRF1 in a fully homogeneous (by ascertained common hepatitis B virus [HBV] positivity) cohort of more HCC patients than previous study. We found that the expression of both UHRF1 mRNA and protein in HCC tumor tissues was significantly higher than that in the matched noncancerous tissues. Furthermore, UHRF1 mRNA expression was higher in HCC cell lines than in the transformed hepatocyte cell line. Additionally, we determined that both UHRF1 mRNA and protein were expressed at significantly higher levels in HCC patients with advanced TNM tumor stage, tumor microemboli, metastasis, and tumor relapse. These results suggested that elevated UHRF1 expression is associated with poor clinicopathologic features in HCC, which is in agreement with previous studies. However, not all patients suffering from tumor metastasis or recurrence had elevated UHRF1 expression, and Cox proportional hazard analysis did not indicate UHRF1 expression as an independent prognostic marker for HCC. We hypothesize that individual differences exist in UHRF1 expression in tumors. To obtain conclusive results, additional studies in larger patient cohorts are required. In addition, survival analysis data demonstrated that patients with elevated UHRF1 expression had shorter relapse-free survival time. Altogether, these results may suggest that elevated UHRF1 expression indicates poor prognosis and promotes tumor progression in HCC.

Previous studies have indicated that UHRF1 promotes the proliferation of tumor cells [24, 41], and downregulation of UHRF1 by RNA interference exerted a growth-promoting effect on tumor cells [42]. In our study, UHRF1 expression was successfully knocked down in HCC cells, and changes of cell characteristics were further explored. Consistent with previous research results, our data showed that the proliferation of HCC cells was significantly inhibited by knockdown of UHRF1. Furthermore, cell death was observed in the later stage of the cell proliferation assay. Using a xenograft model in nude mice, we discovered that tumor growth was significantly inhibited by UHRF1 knockdown, and IHC staining of the subcutaneous tumors showed that Ki-67 protein expression was significantly downregulated in sh-UHRF1-treated mice. Thus, UHRF1 may act as an oncogene by promoting tumor growth in HCC.

In a previous study on gallbladder cancer cells, UHRF1 downregulation induced cell cycle arrest at G1/S transition by inducing p21 in a p53-independent manner [43]. In contrast herewith, other researchers found that depleting cancer cells of UHRF1 causes cell cycle arrest in G2/M phase [44]. In this study, UHRF1 silencing induced G2/M phase cell cycle arrest in HCC cells, which was corroborated by changes in cyclin expression. These findings indicate that UHRF1 may play a critical role during multiple phases of the cell cycle and that the effects of UHRF1 downregulation on cell cycle arrest may be cell type-specific. It is commonly believed that apoptosis evasion plays a critical role in tumorigenesis, and apoptosis has been found to be induced after UHRF1 depletion [32, 43]. However, apoptosis was not induced after UHRF1 knockdown in our study, and surprisingly, non-apoptotic cell death was increased as indicated by fluorescence microscopy, which was consistent with the proliferation assay results. Therefore, we consider that inhibition of cell proliferation in HCC cells was more likely caused by the G2/M phase cell cycle arrest than by apoptosis induced by UHRF1 knockdown.

A considerable body of evidence indicates that hepatocellular EMT is a crucial event in HCC progression, which causes an increase in malignancy of hepatocytes associating with tumor cell migration and invasion [36]. Similar to previous results indicating that tumor aggressiveness was inhibited after UHRF1 silencing [29], our results showed that cell migration and invasion were significantly restrained after UHRF1 knockdown, and changes of EMT markers *in vivo* and *in vitro* indicated that UHRF1 is likely to promote cell migration and invasion by activating EMT. In addition, the regulation of UHRF1 by microRNAs could modulate tumor aggressiveness [31]. Moreover, the mTOR inhibitor Torin-2 could suppress hepatocarcinoma cell growth by downregulating UHRF1 expression [30]. Thus, we believe that UHRF1 may be a promising drug target to prevent recurrence and metastasis.

In conclusion, we revealed that UHRF1 is elevated in HCC and its high expression is significantly correlated with malignant clinicopathologic characteristics and poor prognosis. Furthermore, UHRF1 downregulation can induce G2/M cell cycle arrest and inhibit cell proliferation, tumor aggressiveness, and EMT progression. Thus, UHRF1 plays an important role in HCC and is an attractive cancer target, although further validation is needed.

## MATERIALS AND METHODS

### Ethical approval

The Southern Medical University Ethics Committee approved the protocols according to the Helsinki Declaration (6^th^ revision, 2008) and all patients signed informed consent. All animal protocols were approved by the Institutional Animal Care and Use Committee of Southern Medical University.

### Clinical specimens

Matched HCC tissue specimens including cancerous tissue (CT) and noncancerous tissue (NT) were collected from 102 patients who underwent radical resection in the Department of Hepatobiliary Surgery at Nanfang Hospital of Southern Medical University between November 2010 and November 2014. CT was defined as tissues within 1 cm from the tumor edge without necrosis and NT as tissues exceeding the edge of the tumor by more than 2 cm. Patient inclusion criteria were as follows: (a) no other treatment before surgery; (b) cancerous tissues were pathologically confirmed as HCC; (c) the cut edge was confirmed without residual carcinoma; (d) a complete medical record. The specimens were used for quantitative reverse transcription-polymerase chain reaction (qRT-PCR) and immunohistochemical (IHC) staining.

### Postoperative follow-up

The patients were monitored for prognostic analysis via outpatient examinations or telephone follow-up. Metastasis was defined in patients having distant metastases or visible cancer embolus (in portal vein, biliary tract, or hepatic vein) and those who relapsed within six months. Relapse was defined in patients with characteristic liver cancer lesions detected by imaging examinations (ultrasonic examination, computed tomography scan, or magnetic resonance imaging) and increased AFP level after radical operations. The follow-up ended on August 31^st^, 2015.

### qRT-PCR

RNA was extracted with Trizol, and *UHRF1* and *GAPDH* mRNA expression was measured using the PrimeScript^TM^ First-Strand cDNA Synthesis Kit (TaKaRa, Dalian, China) and the SYBR green qPCR assay (TaKaRa). Primer sequences were as follows: UHRF1 sense primer 5′-CCA GCA GAG CAG CCT CAT C-3′ and antisense primer 5′-TCC TTG AGT GAC GCC AGG A-3′, and GAPDH sense primer 5′-CAG GAG GCA TTG CTG ATG AT-3′ and antisense primer 5′-GAA GGC TGG GGC TCA TTT-3′. The data were analyzed using the 2^-^^△△Ct^ method with *GAPDH* serving as a reference gene for normalization. And we defined cases with expression of UHRF1 mRNA more than median of log2 level of mRNA absolute level in tumor tissues as UHRF1 high expression [21].

### Cell lines

LO2, HepG2, SK-HEP-1, HCCLM3, HEP-3B, MHCC-97L, and MHCC-97H (Institutes for Biological Sciences of the Chinese Academy of Sciences, Shanghai, China) were cultured in high-glucose Dulbecco's modified Eagle's medium (DMEM; Gibco, CA, USA) supplemented with 10% FBS in a humidified atmosphere of 5% CO_2_. Cells in the exponential growth phase were harvested at approximately 80% confluence.

### RNA interference

For transient transfection, UHRF1 silencing was performed in HepG2 and HCCLM3 cells using small interfering RNA (siRNA; GenePharma, Shanghai, China). The siRNA sequences were as follows: siRNA-1 (targeting 5′-TCT CAA CTG CTT TGC TCC CAT CAA T-3′), siRNA-2 (targeting 5′-GCC AGG TGG TCA TGC TCA ACT ACA A-3′), negative control (NC; targeting 5′-TTC TCC GAA CGT GTC ACG TTT-3′). Cells were transfected in 6-well plates for 48 h before use for RNA or protein extraction or for other further assays. Lentiviral-mediated stable transfection with short hairpin RNA (shRNA; GenePharma, Shanghai, China) was conducted according to the manufacturer's protocol. The target and negative control sequences were 5′-GCC AGG TGG TCA TGC TCA ACT ACA A-3′ and 5′-TTC TCC GA A CGT GTC ACG TTT-3′, respectively.

### Cell proliferation assay

Cell proliferation was measured using the CCK-8 assay (Dojindo, Tokyo, Japan) following the manufacturer's protocol. Cells were transfected in 96-well plates with the indicated siRNAs and viable cell numbers were detected at 0 h, 24 h, 48 h, 72 h, and 96 h after transfection.

### Cell cycle analysis

Transfected cells were collected and cell cycle distribution was analyzed using a cell cycle assay kit (BestBio, Shanghai, China) and fluorescence-activated cell sorting (FACS) following the manufacturer's instructions. Cell cycle-related proteins were detected by western blotting.

### Apoptosis assay

Apoptosis was measured by FACS analysis using the Annexin V-FITC/PI Apoptosis Detection Kit (BestBio) according to the manufacturer's instructions.

### Transwell migration and invasion assays

Transfected cells were trypsinized, resuspended in serum-free medium, and transferred to the upper chambers of transwell inserts in 24-well plates (Corning, NY, USA). Migration was induced with 20% FBS medium added to the lower chambers. After 24–48 h, the cells were removed from the chambers, and the inserts were fixed in 4% paraformaldehyde and stained with 0.5% crystal violet. The migrated cells were counted under an inverted microscope (Nikon, Tokyo, Japan). For the invasion assay, all conditions were as described for the migration assay, except that Matrigel-coated inserts were used.

### Western blotting

Cells were lysed on ice with RIPA buffer containing protease and phosphatase inhibitors. Equal amounts of protein from each sample were separated by 10% sodium dodecyl sulfate-polyacrylamide gel electrophoresis (SDS-PAGE) and transferred to polyvinylidene difluoride membranes (Millipore, Bedford, Germany). The membranes were blocked in 5% BSA and incubated overnight at 4°C with primary antibody (1:1000) followed by incubation with HRP-conjugated secondary antibody (1:1000) (Abcam, CA, USA) for 1 h. The immunoblotted proteins were visualized using the Gene-Gnome HR imaging system (Synoptics, UK). The following primary antibodies were used: anti-UHRF1 (Abcam, CA, USA), anti-cyclin B1, anti-cyclin D1, and EMT-related Antibody Sampler Kit (9782s; Cell Signaling Technology, Danvers, MA, USA).

### Animals

Healthy male BALB/c nude mice (SPF grade, 4–5 weeks old, 16–18 g) were purchased from Beijing Vital River Experimental Animal Technology Co., Ltd (Beijing, China). The animals were kept in the Experimental Animal Center of Nanfang Hospital, Southern Medical University. All protocols were approved by the Animal Care Ethics Committee of Southern Medical University.

### *In vivo* experiments

Lentiviral-mediated stably transfected HCCLM3 cells were subcutaneously injected into the flanks of the BALB/c nude mice. A week after injection, the tumor volume was determined for each mouse every four days by measuring two dimensions and was calculated as follows: $\left(V\right)=\text{length}\left(a\right)\times \text{width}{\left(b\right)}^{2}\times \frac{\pi }{6}$. Mouse tumor tissue samples were subjected to hematoxylin and eosin (H&E) and IHC staining.

### IHC staining

Tumor tissue specimens were fixed with 10% neutral formalin for 24–48 h and routinely processed for paraffin embedding. IHC staining was performed as reported previously. UHRF1, Ki-67, E-cadherin, N-cadherin, and vimentin antibodies (1:200) were detected using the streptavidin–peroxidase conjugate method. Immunoreactivity was independently evaluated by two professional pathologists. In the exploration of UHRF1 protein expression in HCC, we defined hepatocytes with light brown- to dark brown-stained nucleus as positive cells, and expression was classified according to the percentage of positive cells as follows: negative expression, <10% positive cells; and positive expression, ≥10% positive cells.

### Statistical analysis

Results are expressed as the mean ± SEM. Significance was established with the SPSS statistical package for Windows Version 21.0 (SPSS, Chicago, USA) and GraphPad Prism 5.0 software (GraphPad Software, Inc., San Diego, CA, USA). Expression of UHRF1 mRNA in HCC was analyzed using Wilcoxon's paired test. The chi-square test was used to examine possible correlations between UHRF1 expression and clinicopathological factors. Disease-specific survival was analyzed using the Kaplan-Meier method. The log-rank test was used to analyze differences in survival. Comparisons among multiple groups were determined using one-way ANOVA. *P* < 0.05 was considered statistically significant.

## Abbreviations

UHRF1: ubiquitin-like with plant homeodomain and ring finger domains, 1
HCC: hepatocellular carcinoma
